# Mapping the corneal thickness and volume in patients with Down syndrome: a comparative population-based study

**DOI:** 10.5935/0004-2749.20200058

**Published:** 2020

**Authors:** Hassan Hashemi, Ali Makateb, Shiva Mehravaran, Akbar Fotouhi, Fereshteh Shariati, Soheila Asgari

**Affiliations:** 1 Noor Ophthalmology Research Center, Noor Eye Hospital, Tehran, Iran; 2 Noor Research Center for Ophthalmic Epidemiology, Noor Eye Hospital, Tehran, Iran; 3 ASCEND Center for Biomedical Research, Morgan State University, Baltimore, MD, USA; 4 Department of Epidemiology and Biostatistics, School of Public Health, Tehran University of Medical Sciences, Tehran, Iran

**Keywords:** Corneal topography, Cornea/pathology, Down syndrome, Diagnostic techniques, Ophthalmological, Comparative study, Topografia da córnea, Córnea/patologia, Síndrome de Down, Técnicas de diagnóstico oftalmológico, Estudo comparativo

## Abstract

**Purpose:**

To measure the central-to-peripheral corneal thickness and its volume according to age and gender in 10-30-year-old patients with Down syndrome (DS) and in matched individuals without DS.

**Methods:**

In the report, 202 normal pattern right eyes of patients with Down syndrome and 190 right eyes of individuals without Down syndrome and compared averages using independent sample t-tests and multiple linear regression models. The measured variables included the apical corneal thickness; the minimum corneal thickness; the average thickness on rings at 2 mm (R2), 3 mm (R3), and 4 mm (R4); the corneal volume in the central zones at 2-, 3-, 4-, and 10-mm diameters; Ambrosio’s relational thickness; and the pachymetric progression indices.

**Results:**

The mean age of the participants was 16.99 ± 4.70 and 17.22 ± 4.54 years (p=0.636). The means ± SD were 516.7 ± 33.0 and 555.7 ± 33.1 µm for apical corneal thicknesses, 508.0 ± 33.5 and 549.0 ± 40.6 µm for minimum corneal thicknesses, 543.0 ± 33.9 and 588.4 ± 33.8 µm for R2s, 584.9 ± 35.6 and 637.0 ± 34.5 µm for R3s, 646.9 ± 38.5 and 707.6 ± 37.1 µm for R4s, 396.4 ± 102.3 and 462.7 ± 96.2 µm for Ambrosio’s relational thicknesses, 1.36 ± 0.37 and 1.22 ± 0.18 for pachymetric progression index maximums, 1.62 ± 0.11 and 1.74 ± 0.11 mm^3^ for corneal volume at 2 mm, 3.73 ± 0.24 and 4.01 ± 0.24 mm^3^ for corneal volume at 3 mm, 6.76 ± 0.44 and 7.30 ± 0.43 mm^3^ for corneal volume at 4 mm, and 57.03 ± 3.44 and 61.51 ± 3.40 mm^3^ for total corneal volume in the Down syndrome and control groups, respectively (all p<0.001). All the above indices were inversely related to age, but not to gender. Ambrosio’s relational thickness maximum and the pachymetric progression index maximum were independent of age and gender.

**Conclusion:**

Non-keratoconic patients with Down syndrome had thin corneas with a homogeneous distribution. Therefore, the reference ranges of cornea thickness and volume should be re-defined for this patient population.

## Abstract (pt)

**Objetivo:**

Medir a espessura corneana central e periférica e se4u volume de acordo com a idade e gênero em pacientes com idades entre 10 e 30 anos com síndrome de Down e em indivíduos saudáveis sem síndrome de Down.

**Métodos:**

No estudo 202 olhos normais direitos de pacientes com síndrome de Down e 190 olhos direitos de indivíduos sem síndrome de Down e médias comparadas usando o teste t de amostras independentes e modelos de regressão linear múltipla. As variáveis medidas incluíram a espessura da córnea apical, a espessura mínima da córnea, a espessura média dos anéis a 2 mm (R2), 3 mm (R3) e 4 mm (R4), o volume corneano nas zonas centrais nos diâmetros de de 2, 3, 4 e 10mm, a espessura relacional de Ambrosio e os índices de progressão paquimétrica.

**Resultados:**

A idade média dos participantes foi de 16,99 ± 4,70 e 17,22 ± 4,54 anos (p=0,636). As médias ± DP foram 516,7 ± 33,0 e 555,7 ± 33,1 µm nas espessuras da córnea apical, 508,0 ± 33,5 e 549,0 ± 40,6 µm para espessura mínima da córnea, 543,0 ± 33,9 e 588,4 ± 33,8 µm nos R2, 584,9 ± 35,6 e 637,0 ± 34,5 µm para R3, 646,9 ± 38,5 e 707,6 ± 37,1 µm para R4, 396,4 ± 102,3 e 462,7 ± 96,2 µm para a espessura relacional de Ambrosio, 1,36 ± 0,37, 1,22 ± 0,18 para os índices máximos de progressão paquimétrico, 1,62 ± 0,11 mm^3^ e 1,74 ± 0,11 mm^3^ para o volume corneano a 2 mm, 3,73 ± 0,24 mm^3^ e 4,01 ± 0,24 mm^3^ para o volume corneano a 3 mm, 6,76 ± 0,44 mm^3^, 7,30 ± 0,43 mm^3^ para o volume corneano a 4 mm e 57,03 ± 3,44 mm^3^ e 61,51 ± 3,40 mm^3^ para o volume corneano total nos grupos com Síndrome de Down e controle, respectivamente (todos p<0,001). Todos os índices acima foram inversamente relacionados à idade mas não ao gênero. A espessura relacional de Ambrosio máxima e o índice de progressão paquimétrico máximo foram independentes da idade e do gênero.

**Conclusão:**

Os pacientes sem ceratocone com Síndrome de Down apresentaram córneas finas com distribuição homogênea. Portanto, os intervalos de referência da espessura e volume da córnea devem ser redefinidos para essa população de pacientes.

## INTRODUCTION

The accurate measurement of corneal thickness at different points has many applications in various ophthalmological fields. The last generation of devices that map the cornea using a tomography approach can accurately show the distribution of corneal thickness from any given point^(1,2)^. In this regard, Ambrosio et al. developed and validated the corneal thickness spatial profile, percentage increase in thickness, pachymetric progression index (PPI), and Ambrosio relational thickness (ART) index to show trends in corneal thickness changes^(3-6)^.

In a population-based study of healthy individuals, the cornea was shown to be thinner at its central thinnest point in 20- to 30-year-old individuals than in those aged under 20 years. In other words, corneal thickness decreases with age in people belonging to this age range^(7)^. A study on 27 children with Down syndrome (DS) aged between 5 and 12 years showed that these patients had a thinner cornea in the center and the thinnest point compared to those of other individuals without DS, and 21% of patients with DS had signs of early keratoconus^(8)^. Thin corneas, and the thickness map, need to be considered when deciding whether to perform refractive surgery.

Therefore, in the present report, we studied the corneal thickness, its distribution, and the corneal volume from the center to the periphery in patients with DS in two groups (10- to 20-year-olds and 20- to 30-year-olds), and we compared the variables to those of ageand gender-matched individuals without DS to provide guidance for diagnoses and treatments in these patient populations.

## METHODS

### Study sample

This report is part of the Down Syndrome Eye Cohort Study, which began in 2016 in Tehran (Iran). We enrolled individuals with DS consecutively from special needs schools and non-governmental organizations dedicated to patients with DS. Selected individuals were invited to the Noor Eye Hospital for an interview and examinations. Also, we enrolled individuals without DS who were candidates for refractive surgery presenting for their first work-up session as well as others presenting for a vision checkup at the Noor Eye Hospital.

The inclusion criteria were ages between 10 and 30 years in both groups and confirmed DS (as stated in the medical records) for the DS group. We excluded individuals with any concurrent genetic disease, such as Klinefelter syndrome, autism, etc., in the DS group and any history of DS and other intellectual disabilities in participants or their families in the group of individuals without DS. In addition, we excluded data from individuals with keratoconus, pterygium, or corneal surgery history. We diagnosed keratoconus based on clinical (Munson’s sign, Vogt’s striae, Fleischer’s ring, apical thinning, or Rizutti’s sign)^(9)^ and paraclinical (maximum keratometry >48D^(10)^, ART at maximum meridians [ART-max] <339 µm^(6)^, inferior-superior asymmetry value >1.4D^(11)^, Belin-Ambrosio Deviation >1.6^(12)^, minimum corneal thickness [MCT] <400 µm^(13)^, and posterior elevation map) findings.

### Examinations

Corneal tomography was performed using the Pentacam HR (Oculus Optikgeräte GmbH, Wetzlar, Ger many) and the Oculus software versions 6.08r27 and 1.21r24. Ocular examinations (both eyes) were con ducted between 8:00 AM and 12:00 AM. Imaging acquisitions were repeated (1 to 3 times in patients with DS) until OK quality statuses were achieved.

In all, we measured the apical corneal thickness (ACT), MCT, the average thickness on rings at 2 mm (R2, average of 12 points at 30° intervals), 3 mm (R3, average of 4 points at 90° intervals), and 4 mm (R4, average of 20 points at 18° intervals) from the center, ART-max, the PPI in the maximum meridian (PPI-max), the corneal volume in the central zones of 2-mm (CV-2 mm), 3-mm (CV-3 mm), and 4-mm (CV-4 mm) diameters, and the total corneal volume in a diameter of 10 mm (total CV).

We derived the ART-max from the MCT divided by the PPI-max. The ART-max shows the ratio of MCT to corneal thickness changes from the periphery to the center in the meridian with the maximum rate of increase. The ART-max is >339 µm in healthy eyes and <339 µm in thin corneas^(6)^.

### Ethical considerations

The Ethics Committee of Tehran University of Medical Sciences approved this project. Prior to entering the study, the methods and objectives of study were explained to the parents of the subjects, and written informed consents were obtained.

### Statistical analysis

Given the high correlation between fellow eyes (lowest= 0.874 with R4 thickness and highest= 0.919 with CV-4 mm), only right eye data were used in the analysis. In the descriptive analyzes, we determined the mean ± standard deviation (SD), 95% confidence interval of the mean, and range of the indices.

The histogram of the distribution of corneal thickness and volume indices show normal distributions, and the Kolmogorov-Smirnov test showed no significant deviations from the normal (all p>0.05).

Independent sample t test was used to compare thickness and volume indices between DS and non-DS groups. Our multiple linear regression model showed concurrent correlations between age, gender and groups, and study indices.

## RESULTS

Of the 250 patients with DS and 200 individuals without DS invited to the study, we included data from 202 patients with DS and from 190 without DS after applying the exclusion criteria (lack of collaboration, concurrent intellectual disability, keratoconus, and pterygium). The mean ages of the DS and non-DS groups were 16.99 ± 4.70 and 17.22 ± 4.54 years (p=0.636), respectively; 75.7% and 78.3% were under 20 years (p=0.547), and 53.0% and 48.7% were male (p=0.396), respectively.

### Corneal thickness

In the DS and non-DS groups, the means ± SDs were 516.7 ± 33.0 and 555.7 ± 33.1 µm for ACT, 508.0 ± 33.5 and 549.0 ± 40.6 µm for MCT, 543.0 ± 33.9 and 588.4 ± 33.8 µm for R2, 584.9 ± 35.6 and 637.0 ± 34.5 µm for R3, 646.9 ± 38.5 and 707.6 ± 37.1 µm for R4, 396.4 ± 102.3 and 462.7 ± 96.2 µm for ART-max, and 1.36 ± 0.37 and 1.22 ± 0.18 for PPI-max, respectively (all p<0.001). We found the ACT to be <500 µm in 29.2% of individuals in the DS group and in 5.2% of those in the non-DS group (p<0.001), and the MCT was <500 µm in 40.1% of individuals with DS and 6.8% in the others (p<0.001).

In addition to DS, age was inversely correlated with the corneal thickness indices, except for the ART-max (p=0.164). The mean indices were higher in the under-20 age group than in the other group (all p<0.05). tables 1 and 2 show the distributions of thickness and progression indices in the age groups of patients with and without DS. The corneal thickness and progression indices showed no significant inter-gender differences (all p>0.05).

**Table 1:** Corneal thickness and volume indices in 10- to 20-years-old Down (n=153 eyes) and healthy (n=148 eyes) subjects

Index	Group	Mean ± SD	CI 95% of mean	Range	P-value^*^
**ACT**	**Down**	518.4 ± 33.9	513.0 to 523.8	452.0 to 646.0	<0.001
	**Normal**	556.6 ± 31.2	551.5 to 561.7	459.0 to 625.0	
**MCT**	**Down**	510.0 ± 34.2	504.4 to 515.3	434.0 to 633.0	<0.001
	**Normal**	549.4 ± 40.9	542.8 to 556.1	231.0 to 622.0	
**Thickness at ring-2mm**	**Down**	544.4 ± 35.0	538.8 to 550.0	477.4 to 677.6	<0.001
	**Normal**	589.3 ± 31.9	584.1 to 594.4	491.4 to 666.7	
**Thickness at ring-3mm**	**Down**	586.0 ± 36.8	584.1 to 591.8	514.2 to 719.7	<0.001
	**Normal**	637.7 ± 32.7	632.4 to 643.0	538.5 to 725.5	
**Thickness at ring-4mm**	**Down**	648.4 ± 39.6	642.1 to 654.7	569.3 to 779.8	<0.001
	**Normal**	708.2 ± 35.3	702.5 to 713.9	605.2 to 822.0	
**ART-max**	**Down**	404.1 ± 99.6	388.1 to 420.0	131.0 to 661.1	<0.001
	**Normal**	465.3 ± 86.1	451.2 to 479.3	290.0 to 850.0	
**PPI-max**	**Down**	1.34 ± 0.35	1.28 to 1.39	0.80 to 3.90	<0.001
	**Normal**	1.22 ± 0.18	1.19 to 1.25	0.70 to 1.70	
**Volume at Diameter-2mm**	**Down**	1.63 ± 0.11	1.62 to 1.65	1.40 to 2.00	<0.001
	**Normal**	1.75 ± 0.11	1.73 to 1.77	1.40 to 2.00	
**Volume at Diameter-3mm**	**Down**	3.74 ± 0.25	3.70 to 3.77	3.30 to 4.70	<0.001
	**Normal**	4.02 ± 0.22	3.98 to 4.06	3.30 to 4.50	
**Volume at Diameter-4mm**	**Down**	6.80 ± 0.44	6.72 to 6.86	5.90 to 8.40	<0.001
	**Normal**	7.31 ± 0.40	7.25 to 7.38	6.10 to 8.30	
**Volume at Diameter-10mm**	**Down**	57.16 ± 3.54	56.60 to 57.73	49.30 to 68.70	<0.001
	**Normal**	61.56 ± 3.22	61.04 to 62.08	52.70 to 71.50	

ACT= apical corneal thickness; MCT= minimum corneal thickness; ART-max= Ambrosio’s relational thickness at maximum meridian; PPI-max= pachymetric progression index in the maximum meridian.

^*^ Related to comparison of mean ± SD indices between two groups.

**Table 2:** Corneal thickness and volume indices in 21- to 30-years-old Down (n=49 eyes) and healthy (n=41 eyes) subjects

Index	Group	Mean ± SD	CI 95% of mean	Range	P-value^*^
**ACT**	**Down**	511.2 ± 29.4	502.8 to 519.7	459.0 to 582.0	<0.001
	**Normal**	552.0 ± 39.7	539.5 to 564.5	480.0 to 639.0	
**MCT**	**Down**	502.3 ± 30.9	493.4 to 511.2	434.0 to 575.0	<0.001
	**Normal**	546.9 ± 40.5	534.1 to 559.7	472.0 to 636.0	
**Thickness at ring-2mm**	**Down**	538.8 ± 30.4	530.0 to 547.5	480.5 to 622.0	<0.001
	**Normal**	584.6 ± 40.7	571.8 to 597.5	509.1 to 666.5	
**Thickness at ring-3mm**	**Down**	581.6 ± 31.6	572.6 to 590.7	522.2 to 668.7	<0.001
	**Normal**	633.3 ± 40.9	620.4 to 646.3	557.7 to 716.0	
**Thickness at ring-4mm**	**Down**	642.1 ± 34.8	632.1 to 652.1	567.5 to 739.7	<0.001
	**Normal**	704.0 ± 43.3	690.3 to 717.6	624.1 to 796.3	
**ART-max**	**Down**	379.1 ± 99.8	350.4 to 407.8	128.0 to 567.8	<0.001
	**Normal**	464.3 ± 107.5	430.4 to 498.2	316.9 to 795.0	
**PPI-max**	**Down**	1.42 ± 0.44	1.30 to 1.55	0.90 to 3.50	0.005
	**Normal**	1.22 ± 0.20	1.16 to 1.28	0.80 to 1.60	
**Volume at diameter-2mm**	**Down**	1.60 ± 0.09	1.58 to 1.63	1.40 to 1.80	<0.001
	**Normal**	1.73 ± 0.12	1.69 to 1.77	1.50 to 2.00	
**Volume at diameter-3mm**	**Down**	3.69 ± 0.21	3.63 to 3.75	3.30 to 4.20	<0.001
	**Normal**	3.98 ± 0.29	3.89 to 4.07	3.50 to 46.00	
**Volume at diameter-4mm**	**Down**	6.71 ± 0.38	6.61 to 6.82	6.00 to 7.70	<0.001
	**Normal**	7.25 ± 0.51	7.08 to 7.41	6.30 to 8.30	
**Volume at diameter-10mm**	**Down**	56.61 ± 3.11	55.72 to 57.50	50.20 to 65.40	<0.001
	**Normal**	61.19 ± 4.04	59.92 to 62.46	53.60 to 70.60	

ACT= apical corneal thickness; MCT= minimum corneal thickness; ART-max= Ambrosio’s relational thickness in the maximum meridian; PPI-max= pachymetric progression index in the maximum meridian.

* Related to comparison of mean±SD indices between two groups.

The MCTs were located at 1.06 ± 0.37 and 0.81 ± 0.33 mm from the apex in the DS and non-DS groups, respectively; this difference was statistically significant (p<0.001). The thinnest point was in the inferotemporal quadrant in 96.9% of the patients with DS and in the superotemporal quadrant in 98.4% of those without DS (p<0.001).

### Corneal volume

**Figure 1:**
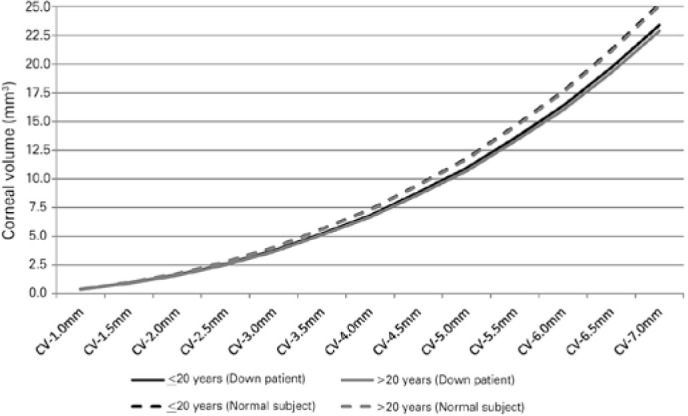
Central-to-peripheral corneal volume in patients with Down syn drome (DS) (n=202 eyes) and in individuals without DS (n=190 eyes) based on the age groups.

The means ± SDs were 1.62 ± 0.11 and 1.74 ± 0.11 mm^3^ for CV-2 mm, 3.73 ± 0.24 and 4.01 ± 0.24 mm^3^ for CV-3 mm, 6.76 ± 0.44 and 7.30 ± 0.43 mm^3^ for CV-4 mm, and 57.03 ± 3.44 and 61.51 ± 3.40 mm^3^ for total CV in DS and non-DS groups (all p<0.001), respectively. All corneal volume indices were significantly associated with age (all p<0.05), and values were higher in the under-20 age groups than in the over-20 age groups (Tables 1, 2). But no significant difference was observed between the two genders (all p>0.05). Figure 1 shows the corneal volume index distributions from the corneal center to the periphery.

## DISCUSSION

DS is a common chromosomal abnormality with ocular manifestations. Since the screen superoxide dismutase 1 gene, a candidate gene for keratoconus, is located on chromosome 21^(14,15)^, it has been suggested that patients with DS are more susceptible to keratoconus. There are contradicting reports concerning the corneal thickness in this patient population. Some studies with results similar to ours have reported thinner corneas in patients with DS than in individuals without DS^(8,16)^, but some have rejected this finding: Karadag et al.^(17)^ reported that the cornea is thicker in the center in 17-55-year-old subjects with intellectual disability, including individuals with DS, compared with that in those without DS. Akinci et al.^(18)^ also studied a 5- to 17-year-old sample of patients with DS, and they reported that the central cornea was thicker than normal in them. Since these two studies included individuals with different syndromes, we cannot provide accurate comparisons of their findings with ours. If patients with DS have thinner corneas than normal, they need to be discriminated from ectatic corneas. Therefore, in this report, in addition to corneal thickness and volume, we investigated corneal thickness patterns in a 10-30-year-old sample of patients with DS.

The mean ACT in our study was 518.4 µm in patients with DS under 20 years. The mean was 502.3 µm in the study by Aslan et al. in 5- to 13-year-olds (measured with Pentacam)^(8)^, 488.4 µm in the study by Evereklioglu et al. in 5- to 15-year-olds (measured with ultrasound)^(19)^, and 480 µm in the study by Haugen et al. in 14- to 26-year-olds (measured with the Nidek Model EAS)^(16)^. Given the various measurement techniques, comparisons among these studies can be imitated, but when comparing our findings to those in the study by Aslan et al. (both with Pentacam), we find that Iranian children with DS have thicker corneas than Turkish children with DS. We observed a similar difference when comparing our MCT measurements (510.0 µm) to those in the study by Aslan et al. (487.8 µm); the thinner corneas in their study^(8)^ may be attributed to a keratoconus rate of 21% in their patients with DS and of 1.3% in their other patients.

To our knowledge, no studies have measured corneal thicknesses of 20- to 30-year-old patients with DS. Comparing ACTs and MCTs in this population (511.2 and 502.3 µm, respectively) with those in the non-DS population (552.0 and 546.9 µm, respectively) also suggests thinner corneas for 20- to 30-year-old patients with DS in the apex and the thinnest point compared to those in individuals without DS.

However, the corneal thickness distribution indices (ART-max and PPI-max) indicate that the increase in the corneal thickness from the center to the periphery and the ratio of the MCT to this progression are similar in under-20- and over-20-year age groups, and a homogeneous pattern is observed in both groups. In other words, age-related total corneal thinning was not obser ved in these patients. This is important because the 20- to 30-year age group forms a major proportion of the population of refractive surgery candidates.

In this study, the total corneal volume in the group of patients under 20 years was 57.0 mm^3^, which was higher than that reported in the study by Aslan et al. (56.2 mm^3^)^(8)^. In our study, the distribution of corneal volume from the center to the periphery showed a homogeneous distribution for corneal thickness. As demonstrated in figure 1, the slope of increase in the corneal volume was similar in the two age groups (under and over 20 years).

The strength of this study compared to others is its larger sample size in both age groups. In addition, since the individuals in the sample had normal eyes free of any corneal pathology, such as keratoconus or pterygium, the normal range obtained can be used as a guide for clinicians. Also, the corneal thickness distribution, as oppo sed to focal measurements, can be helpful, especially for patients with DS undergoing refractive surgery.

In conclusion, patients with DS appear to have thinner corneas and the normal range of these parameters are lower than in individuals without DS^(20,21)^. As such, both the upper and lower confidence limits of the corneal thickness are lower in patients with DS. Also, thickness values >500 µm and ART-max values >339 µm can be observed in patients without ectasia signs. Thus, the normal range and patterns of thickness change from the center to the periphery should be considered when diagnosing eye diseases such as keratoconus.
